# A music- and game-based oral health education for visually impaired school children; multilevel analysis of a cluster randomized controlled trial

**DOI:** 10.1186/s12903-020-01131-5

**Published:** 2020-05-18

**Authors:** Nasrin Sharififard, Katayoun Sargeran, Mahdia Gholami, Farid Zayeri

**Affiliations:** 1 Research Center for Caries Prevention, Dentistry Research Institute, Department of Community Oral Health, School of Dentistry, Tehran University of Medical Sciences, Tehran, Iran; 2 Department of Community Oral Health, School of Dentistry, Tehran University of Medical Sciences, Tehran, Iran; 3 Proteomics Research Center and Department of Biostatistics, Faculty of Paramedical Sciences, Shahid Beheshti University of Medical Sciences, Tehran, Iran

**Keywords:** Oral health, Visually impairment, Game-based education, Music-based education, School children, Iran

## Abstract

**Background:**

Visually impaired children encounter some challenges for their oral health. We aimed to compare the effectiveness of oral health education using Audio Tactile Performance (ATP) technique alone, ATP combined with oral health education for mothers, and ATP along with art package on the oral health status of visually impaired children.

**Materials and methods:**

This cluster, randomized trial, included visually impaired children from preschool to 10th grade (32 classes, *n* = 200), in Tehran, Iran, in 2018–2019. A questionnaire was filled out through face to face interviews at baseline regarding age, gender, status of visual impairment, and place of residence. The Simplified Oral Hygiene Index (OHI-S) and Bleeding on Probing (BOP) were examined afterward. Classes were randomly assigned to three groups through simple randomization: 1. Art group (ATP, game-based, and music-based education (11 classes, *n* = 66); 2. Mothers group (children received ATP and their mothers received education by telephone (10 classes, *n* = 73); and 3. Control group (children received ATP (11 classes, *n* = 61). Children received reinforcement after 1 and 2 weeks. Follow-up oral examinations were performed after 1 and 2 months by a blind calibrated examiner. Descriptive statistics were performed using Chi-Square, and ANOVA tests by SPSS (version 22). In analytic statistics, two-level mixed-effects models for BOP and OHI-S were fitted using the Statistical Analysis Software (SAS) version 9.4. Both models fitted with the grouping variable and time (baseline, 1, and 2 months after interventions) as the fixed effects.

**Results:**

The mean age (SD) of the children was 12.29 (3.45) years (range: 6–17). Male children (67%) more than female children (33%) participated in the study. Also, 35.5% were blind, and 12% resided at the dormitory. The art and mothers groups had no statistically significant difference compared with the control group, in terms of OHI-S (*P* = 0.92, and 0.39, respectively) and BOP (OR = 0.64, and 0.66, respectively). The time effect was statistically significant in both BOP and OHI-S models (*P* < 0.0001).

**Conclusions:**

ATP technique is an effective method to improve the oral health status of visually impaired school children. Oral health promotion programs can be done using this method to tackle oral hygiene problems in visually impaired children.

**Trial registration:**

(https://www.irct.ir/trial/34676: Nov 29th, 2018)

## Background

Visual impairment is an important public health problem worldwide [1]. Globally, about 2.2 billion people suffer from a visual impairment or blindness. Among children, congenital cataract and retinopathy of prematurity are the leading causes of vision impairment in low-income and high income countries respectively [2]. In Iran age-standardized prevalence of vision loss was 12.3% in 2015 [3].

Studies show that poor oral hygiene [4–6], dental caries [4–9], various levels of periodontal diseases [4, 7–10], trauma to the anterior teeth [6, 9, 11], and hypoplastic teeth [11] are frequent in visually impaired children. Poor oral health in these children may be caused by different factors including lack of knowledge and behavior about oral hygiene [4], infrequent dental visit [4, 6, 7], difficulty to access oral care facilities, and neglection of parents and health providers while the focus is kept predominantly on managing their existing disability [4, 12].

Some difficulties such as the lack of hand-eye coordination in visually impaired children can affect the quality of plaque removing. Also, they lack the ability to visually assess whether visible dental caries or gingival bleeding exist during tooth brushing, which consequently affects the oral health status and early dental visit [13].

Visually impaired children depend more on sound, smell, touch, taste, and speech to orient themselves to a situation [12, 14]. Such children have an equal right to receive the proper oral health education and motivation [15, 16]. Studies have demonstrated improvement in oral hygiene following oral health education among visually impaired children [12, 17–22]. Various methods of oral health education were used for visually impaired children to promote their oral health. Most of the studies revealed that multisensory education is more effective than unisensory methods [12, 18, 19, 22]. Educating the brushing method through Audio Tactile Performance (ATP) [17, 20] involves auditory and tactile senses, which are more powerful in visually impaired people. Using educational casts and music-based education were also effective for visually impaired children [21]. Games are desirable learning modes, as they can make oral health education more interesting and effective for children [23, 24]. However, to our knowledge game-based education, has not been used in the oral health education of visually impaired children.

In addition, mothers of visually impaired children, accompany their children more than mothers with normal children [25]. Also, they face difficult aspects of child care, access to health care, overloading due to the child dependency, and lack of support within their own families [26]. Moreover, we lack studies to assess the role of mothers in the oral health of visually impaired children. Consequently, a randomized controlled trial was conducted to compare different oral health educational methods to find the most effective ones for these group of children. Thus, the aim of the present study was to compare the effectiveness of oral health educational methods using ATP technique alone, ATP combined with oral health education for mothers, and ATP and art package on the oral hygiene status of children with visual impairment.

## Methods

### Trial design

The present study was a double-blind, parallel, cluster, randomized trial in 6–17-year-old visually impaired school children in Tehran, Iran. Study procedures consisted of a pilot, baseline study, interventions, two reinforcements for allocated groups, and follow-up examinations after 1 and 2 months. The total study period was from December 2018 to April 2019.

### Behavioral changes theory

The design and planning of this study were based on the modified PRECEDE-PROCEED oral health promotion model. PRECEDE (Predisposing, Reinforcing, and Enabling Constructs in Educational Diagnosis) highlights a diagnostic planning process to develop the targeted public health programs. PROCEED (Policy, Regulatory, and Organizational Constructs in Educational and Environmental Development) outlines the implementation and evaluation of the intervention designed in the PRECEDE component [27, 28]. The different parts of the model in the present study are following: 1- Fluoride therapy is routinely provided for the 1st to 6th grades by the Iranian Ministry of Health, as a national program, twice a year in all schools (it is considered as an environmental factor); 2- The educational level of mothers is an enabling factor; 3- children’s oral health conceptions are predisposing factor; and 4- Mothers’ oral health knowledge, attitude, and behavior as well as supervision of child’s tooth brushing are considered as reinforcing factors [28].

### Study population and randomization (allocation concealment mechanism)

There were three schools - one school for girls (*n* = 97) and two schools for boys (*n* = 150 and 53) - for visually impaired children in Tehran. Total of 300 children were assessed for eligibility to participate in the study. Inclusion criteria were being in preschool to 10th grade (aged 6–17 years) and having parents’ consent. Thus 214 children of 300, in 32 classes met the inclusion criteria (only three children did not have parents’ consent). Children were excluded from the study if they were not cooperative, and/or had mental or other physical disabilities or were absent in the baseline study. Consequently, data collection was performed for 200 children at baseline.

After baseline study, classes were considered as the units of randomization. In each school, stratification was done among the classes (clusters), and it creates two layers: preschool to 5th grade; 6th to 10th grade. Each class and each group was named by a Latin code. Within each layer in each school, simple randomization by Microsoft Excel was done by a colleague who was blind to the study. So, each class was randomly entered into one of the three study groups (control group, mothers group, art group). Consequently, eligible participants were 200 in 32 classes, which were divided into three groups: control (11 classes, *n* = 61), mothers (10 classes, *n* = 73), and art (11 classes, *n* = 66).

### Blinding

This trial was a double-blind study regarding the outcome measure assessment and data analysis. The examiner who conducted the post-intervention oral examination was blind to the group allocation of the study participants. Statistical analysis was conducted by the trial statistician, who was blind to the allocation, i.e. the intervention groups were coded without disclosing the labeling.

### Data collection

#### Outcome measurement

Outcome measures included improvement in oral hygiene status measured through a decrease in the simplified oral hygiene index (OHI-S) and bleeding on probing (BOP). The OHI-S described by J. Greene and J. R. Vermillion in 1964 [29] and consisted of debris index (DI) and calculus index (CI). Six tooth surfaces are scored: (3, 8, 14,19, 24, and 30), and in the permanent dentition or (A, E, F, K, O, and P) in the primary dentition on a scale of 0 to 3. The debris scores were added together and divided by the number of examined tooth for each person to calculate DI. The same process was used to obtain CI [29, 30]. The sum of DI and CI were defined as OHI-S. In this study OHI-S was considered as a quantitative outcome variable. The examination for BOP proceeded from the first to the fourth quadrants using the WHO Periodontal Probe. Buccal and mediobuccal were examined for the presence or absence of bleeding. Bleeding on less than 30% of all probed sites for each person, was considered as local and bleeding on 30% of sites or higher was defined as general bleeding [31]. For analysis, local and general bleeding were coded “YES” and absence of bleeding was coded “NO”. Baseline examination was done by the first examiner and post-intervention examinations were performed by a blind calibrated examiner.

#### Pilot study and calibration

For pilot study, we asked students from the 11th grade to participate in the study to avoid losing eligible study population of children in the preschool to 10th grade. First the two examiners reviewed how to examine for BOP and OHI-S. They examined one volunteer teacher to observe the performance of each another and discuss agreement on coding.

For intra-examiner calibration, each examiner examined 10 children for BOP assessment in separate classes. The second assessment was done after half an hour to resolve previous bleeding (Kappa for BOP = 1). They also assessed DI and CI for OHI-S. As due to removing the debris by explorer during the first examination, it was not possible to assess DI again, only for CI, intra-class correlation coefficient (ICC) was calculated (ICC = 1).

For inter-examiner calibration, 20 children were examined by both examiners. The first examiner assessed BOP, half an hour later the second examiner assessed BOP again (Kappa for BOP = 1). For DI index, in each child, one of the examiners collected debris by explorer, then according to the amount of debris on explorer, both recorded the score in separate assessment forms (ICC for DI = 0.88). For CI they examined children separately (ICC for CI = 0.95). In total, ICC for OHI-S was calculated 0.89.

WHO questionnaire for children [32] including oral health-related questions and demographic variables regarding age, gender, grade, status of visual impairment, place of residence, father’s education and mother’s education was filled out through face to face interview by one of the researchers (NSh). An oral health education, using ATP technique and art package was performed subsequently. The results of the pilot study were discussed among the research team members and minor revisions were performed to the study protocol where needed. The main revision was for brushing technique. At first, scrub for 6–9 and modified bass for 10–17- year-old children had been considered [33–39]. However, in the pilot study we found that modified bass was confusing for some children. To achieve the desired benefits of oral health education, it is essential to consider individual training according to the developmental stage and motor skills of each child [37]. As there is no statistical difference between the tooth brushing techniques [38] modified bass was replaced by roll technique, which was simpler.

#### Baseline

The WHO questionnaire for children was filled as same as the pilot study for the participants (*n* = 200) in 32 classes. According to the 11th revision of International Classification of Diseases (ICD-11), a classification of severity of vision impairment consists of six levels: no vision impairment; moderate vision impairment; severe vision impairment; and three categories for blindness [40]. In this study, obtained from the school records, visual impairment was categorized into “blind” and “low vision” [7]. Moderate and severe vision impairment were grouped under the term “low vision” and three categories for blindness were considered “blind” [9]. Oral examinations were implemented to assess OHI-S, and BOP. The school children were examined at their respective classes, seated on an ordinary chair, under proper illumination of a headlamp, by a disposable mouth mirror, explorer, and WHO periodontal probe while taking protective cross infection control measures using disposable gloves and masks.

#### Interventions

Classes (clusters) were randomly assigned to three groups: 1- Art group (children received education by ATP and art package); 2- Mothers group (children educated by ATP and their mothers received education on phone); 3- Control group (children received education through ATP). To prevent the contamination between the art group with other groups, interventions for the art group were performed after the end of the interventions and follow-ups of the other two groups.

##### ATP (audio tactile performance)

Students in all groups received education through the ATP method. Audio: A verbal oral health education was delivered to the children regarding the importance of teeth and dental caries and gingival health. Then oral hygiene instructions were delivered: 1. Tooth brushing twice daily, all surfaces of the tooth (morning and before bed) for two minutes; 2. Minimal wash after tooth brushing to keep fluoride; 3. Rinsing after main meal and snacks; 4. Restricting the consumption of sugar. Tactile: We asked each child to show his/her own tooth brushing method with a toothbrush. Performance: The proper brushing technique was taught individually by the intra-oral guidance of the children’s hand with their own toothbrush until it was ensured that the student could properly handle it. Tooth brushing was demonstrated through the scrub technique for 6–9-year-olds and roll technique for 10–17-year-old children [33–39].

##### Education for mothers through telephone

Oral health instructions were provided to mothers. The instructions included the importance of oral hygiene to prevent caries and gingival disease, the role of fluoride in controlling caries, the necessity of controlling the consumption of sugar and the importance of regular dental visits. The mothers were then asked to perform the instructions mentioned above for themselves and encourage children to do accordingly. However, explaining the brushing method on phone for mothers was not practical, thus we just asked them to remind children to brush twice daily for 2 min in the way we taught them at school.

##### Art package

This package consisted of a game-based and music-based education. First, the children were asked to move their fingers over the educational dental casts to distinguish the cavities, calculus, and gingival swelling. Next, they made handcrafts about mouth and teeth using play dough. A piece of 2-min music was prepared focused on the direction of tooth brushing for each jaw, right and left sides and tooth surface. The music was played in the class and students performed the tooth brushing while listening to the music until they were able to brush properly. The music was given to them on CD and through a social medium to be used at home at brush time.

##### Reinforcement

1 week and 2 weeks after the beginning of the intervention for each class, the instructions were repeated and the questions were answered and the problems were addressed.

### Statistical analysis

Statistical analyses were conducted by the trial statistician, who was blind to the allocation, i.e. the treatment groups were coded without disclosing the labeling. Intention to treat (ITT) and analysis of available data were applied. The SPSS software version 22 (IBM, Armonk, NY, USA) and the Statistical Analysis Software (SAS) version 9.4 were used for data analysis. The descriptive part of the statistical analysis was performed using Chi-Square, and one-way ANOVA by SPSS.

In the analytic part of the statistical analysis, two-level mixed-effects models were used to account for the heterogeneity among school children (level 1) and classes (level 2) using the SAS (GLIMMIX procedure). For the OHI-S variable (as the continuous response), a linear two-level mixed-effects model was fitted. In addition, for the BOP variable (as the binary response), a binary logistic two-level mixed-effects model was used. In these two models, we first included the group, time, and group * time effects. Then models were fitted, only with the group and time variables as the fixed effects. *P*-values less than 0.05 were considered statistically significant.

### Ethics statement

Ethical clearance was sought from the Ethics Committee of Tehran University of Medical Sciences (IR.TUMS.DENTISTRY.REC.1397.104). Written informed consent was obtained from parents before the study. In addition to the informed consent by the parents, the children also gave “assent”. Researcher talked to them in a language that they could understand, to be sure they were happy to participate in the study.

## Results

**Fig. 1:**
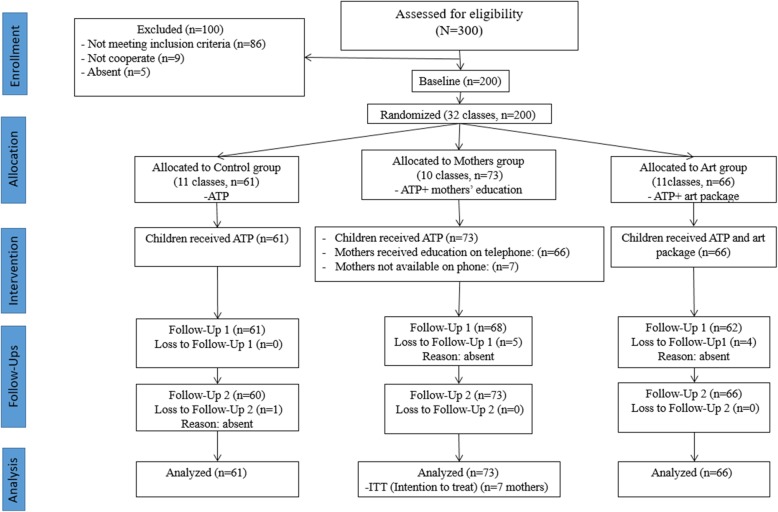
CONSORT 2010 flow diagram of visually impaired school children’s recruitment and follow-ups

A total of 200 participants (32 classes) were included in the final analysis. Response rate of the participants was 95% in the first follow-up and 99% in the second follow-up. ITT strategy was applied for seven mothers who were not available on phone. The loss to follow-up children that we had in each follow-up, were present in the other follow-up examination and analysis of available data was fitted. A diagrammatical representation of the trial according to CONSORT [41] is provided in Figure 1.

Baseline demographics information and clinical oral status are illustrated in Table 1. The mean age (SD) of the children was 12.29 (3.45) years, ranged from 6 to 17 years. More male (67%) than female children (33%) participated in the study. Blind children comprised 35.5% of the respondents and the remaining children had low vision. The incidence of BOP was observed in 69.7% and the mean (SD) OHI-S was 1.99 (0.69). Stratification by grade in two layers (preschool to 5th grade; 6th to 10th grade) before cluster randomization helped to have more homogeneity in groups. Despite unequal number of each stratum in 3 groups, there was no statistically significant difference among groups regarding grade (*P* = 0.12).

**Table 1:** Characteristics of the study participants at baseline (*n* = 200)

	Control *n* = 61	Mothers *n* = 73	Art n = 66	Total n = 200	*P*-values Among three groups
Variable	N (%)	N (%)	N (%)	N (%)	Chi-Square
Gender					
Male	50 (82)	40 (54.8)	44 (66.7)	134 (67)	0.004^a^
Female	11 (18)	33 (45.2)	22 (33.3)	66 (33)	
Age					
6–12	31 (50.8)	30 (41.1)	24 (36.4)	85 (42.5)	0.25
13–17	30 (49.2)	43 (58.9)	42 (63.6)	115 (57.5)	
Grade					
Preschool- 5th grade	31 (50.8)	26 (35.6)	23 (34.8)	80 (40)	0.12
6th- 10th grade	30 (49.2)	47 (64.4)	43 (65.2)	120 (60)	
Place of residence					
Dormitory	10 (16.4)	14 (19.2)	20 (30.3)	44 (22)	0.13
Home	51 (83.6)	59 (80.8)	46 (69.7)	156 (78)	
Status of visual impairment					
Blind	23 (37.7)	25 (34.2)	23 (34.8)	71 (35.5)	0.91
Low vision	38 (62.3)	48 (65.8)	43 (65.2)	129 (64.5)	
BOP					
Yes	51 (83.6)	52 (71.2)	46 (69.7)	149 (74.5)	0.14
No	10 (16.4)	21 (28.8)	20 (30.3)	51 (25.5)	
	**Mean (SD)**	**Mean (SD)**	**Mean (SD)**	**Mean (SD)**	**ANOVA**
OHI-S	2.02(0.68)	2.00 (0.70)	1.95 (0.68)	1.99 (0.69)	0.84
Fathers’ education (year)^b^	10.35 (5.20)	11.67 (4.43)	9.76 (4.68)	10.66 (4.80)	0.07
Missing (*n* = 16)					
Mothers’ education (year)^b^	9.79 (4.80)	11.20 (4.17)	8.11 (4.07)	9.78 (4.51)	< 0.001^a^
Missing (*n* = 11)					

*Abbreviations: BOP* Bleeding On Probing, *OHI-S* Oral Hygiene Index-Simplified

^a^statistically significant, ^b^the number of years at school or university

**Fig. 2:**
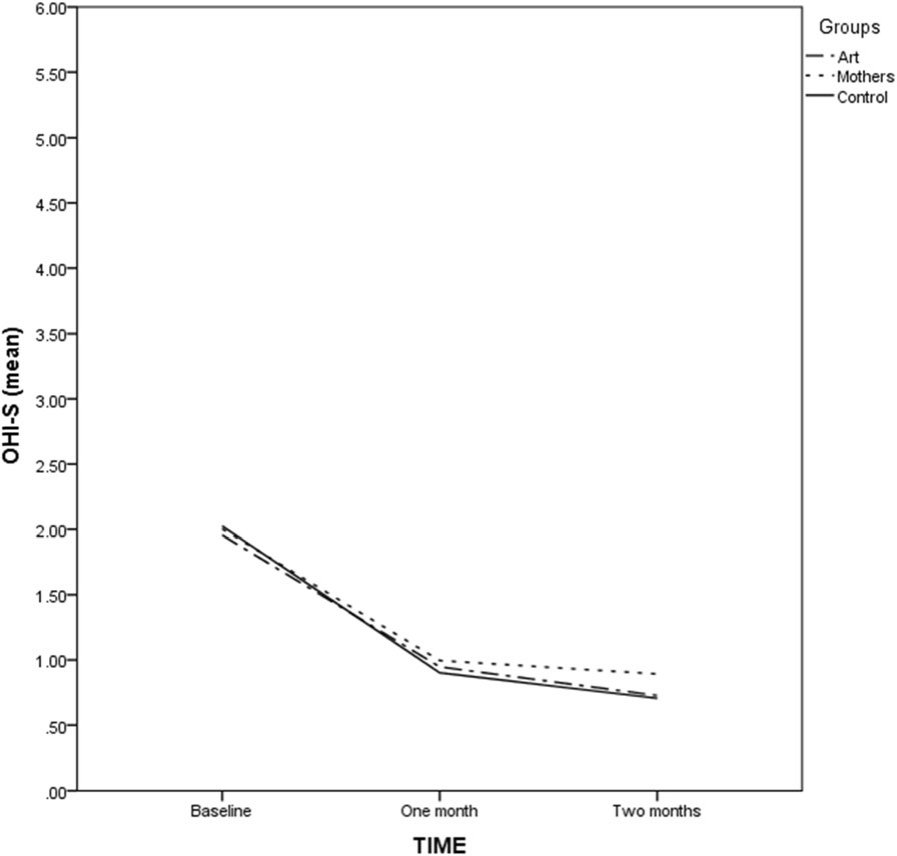
Mean OHI-S in the three groups over time (*n* = 200)

Figure 2 shows the graphical representation of mean OHI-S in the three groups over time. Mean OHI-S in the control group decreased from 2.02 at baseline to 0.90 and 0.71 at 1 month and 2 months follow-ups, respectively. In the art group, mean OHI-S decreased from 1.95 at baseline to 0.95 and 0.73 at follow-ups, respectively. In the mothers group, mean OHI-S improved from 2 at baseline to 0.99 and 0.89 at follow-ups, respectively.

**Fig. 3:**
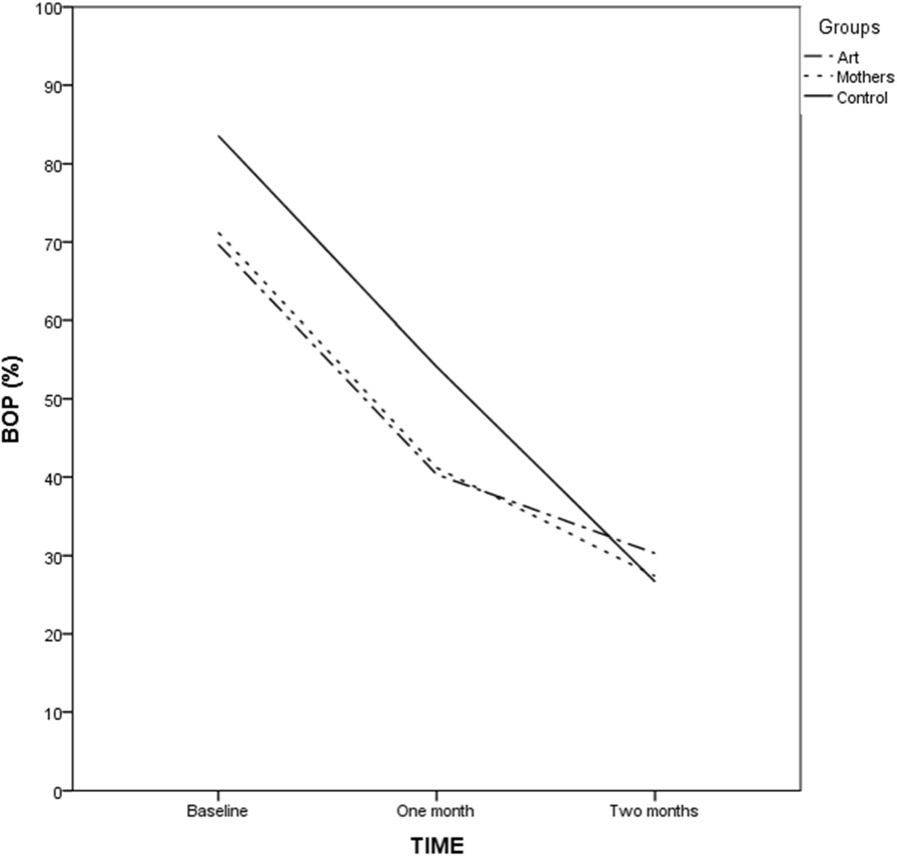
BOP percentages in the three groups over time (*n* = 200)

Figure 3 illustrates the graphical percentages of BOP in the three groups over time. One month after intervention, BOP decreased in the control group from 84 to 54%, whereas it decreased in the art group, from 70 to 40% and in the mothers group from 71 to 41%. After the first follow-up examination, decreasing of BOP continued in all groups. Consequently, 2 months after the intervention, BOP in the control, art, and mothers groups reached 27, 30, and 27%, respectively.

In both BOP and OHI-S two-level mixed-effects models, the interaction between group and time effects was not statistically significant. Consequently, results were shown in models fitted only with the group and time variables as the fixed effects. Tables 2 and 3 depict the estimates from fitting two models, responses for OHI-S and BOP. As can be seen, the group effect was not statistically significant in both models. This means that the three groups had no statistically significant difference in terms of mean OHI-S and odds of BOP.

**Table 2:** Estimates from fitting two-level mixed-effects model for OHI-S in the study participants (n = 200)

Variable	Category	EST	SE	95%CI	*P*-values
Group	Art	0.01	0.11	−0.21, 0.23	0.92
	Mothers	0.09	0.11	−0.13, 0.31	0.39
	Control	Ref			
Time		−0.61	0.02	−0.65, − 0.57	<.0001^a^

*Abbreviations: EST* Estimate, *SE* Standard Error, *Ref* reference category

^a^statistically significant

**Table 3:** Estimates from fitting two-level mixed-effects model for BOP in the study participants (n = 200)

Variable	Category	EST	SE	*P*-values	OR	95% CI
Group	Art	−0.44	0.43	0.31	0.64	0.28, 1.49
	Mothers	−0.42	0.43	0.33	0.66	0.28, 1.52
	Control	Ref				
Time		−1.27	0.13	<.0001^a^	0.28	0.22, 0.36

*Abbreviations: EST* Estimation, *SE* Standard Error, *OR* Odds Ratio, *Ref* reference category

^a^statistically significant

However, the time effect was statistically significant in both models (*P* < 0.0001). In these models, one can differently interpret the time effect. In the model with OHI-S as the response variable, the estimate − 0.61 for the time effect shows that passing each month resulted in about 0.61 unit decrease in mean OHI-S values (Table 2). In addition, the estimate of − 1.27 for the time effect in model with BOP as the response tells us that passing each month has led to about 72% decrease in the odds of incidence of BOP (OR = 0.28) (Table 3).

## Discussion

To the best of our knowledge, no available published data has investigated the role of mothers and game in improving oral hygiene in visually impaired children. Since, there was oral health improvement in all the three groups with no statistical significant difference among them, ATP technique, which was served in all groups, is an effective oral health education method to improve the oral health status of visually impaired school children in the short term. It was in line with Hebbal’s study that showed pre- and post-education through ATP decreased mean plaque scores significantly (*p* < 0.001) [17]. A verbal explanation with friendly communication encouraged the students to cooperate. Hand over hand guidance to teach brushing provided a proper tactile sense and positive emotional feeling. Their perfect consequent performance of tooth brushing made them confident to do it at home.

Our findings did not reveal any differences among the groups that are in line with some other studies. Gautam and colleagues [22] showed that in 6–16-year-old visually impaired children, there was a decrease in the mean plaque scores at all-time intervals after a different combination of audio, Braille, and tactile education methods in individual groups compared with the baseline but there was no difference among the groups (group A: audio aids + Braille, group B: audio aids + tactile models, and group C: audio aids + Braille + tactile models) after one month. Mahantesha and co-workers [15] confirmed that even though both oral hygiene instructions by audio recordings and written instructions in Braille showed a decrease in the plaque score, inter-group comparison of Patient Hygiene Performance (PHP) index score was not statistically significant after three months among 6–20-year-old visually impaired children. The findings of the study done by Shetty and others showed improvement in gingival health status and oral hygiene status after using a specially designed educational model with music and the cooperation of caretakers [21].

Although the children have enjoyed game and music-based education at class time, most of them gave negative feedback in the reinforcing sessions that brushing without music was easier to do at home. It indicates that ATP method is more feasible and effective for visually impaired children.

To our knowledge, this study is the first of its kind to evaluate mothers’ role in oral health promotion of visually impaired children. Although in the present study, educating mothers made no statistically significant difference in terms of mean OHI-S and odds of BOP, it is recommended to design similar studies with a larger sample size and longer follow-ups for further evaluation of the mothers’ role.

Tehran is the multicultural capital of Iran due to large immigration from different areas of the country. Thus, our study findings can be generalized to all schools for visually impaired children in Iran. However further studies at international level should examine whether these findings have wider generalizability. Having considered the explanation of the methods in details, a suitable applicability is expected for the present study.

### Strengths

One of the strengths of the present study was the high response rate of the participants (95–99%). In addition, all three schools for visually impaired children in Tehran were included. Moreover, Stratification by grade before cluster randomization is another strength, which helped to have more homogeneity in the groups regarding the grade. Blinding in this trial, regarding the outcome measure assessment and data analysis, could reduce the risk of biases. Consequently, we believe that the findings of this study have an acceptable internal validity.

### Limitations

In terms of limitations of the present study, the research team have recognized the potential bias that might have been caused by the presence of unequal number of children in classes. Randomization among classes instead of individuals, helped to control the interventions’ contamination. However, unequal number of children in classes, might cause non-homogeneity (random bias) according to gender among the groups (Table 1). Furthermore, School time schedule limited us to have longer follow-ups. This might be considered as another limitation of our work. In addition, in the mothers group, 14 children of 73, resided at the dormitory. Despite, we asked their mothers to call every day and encourage them to use oral health instructions, these children lost the opportunity to have the face to face daily advice of their mothers.

## Conclusion

In the present study, no statistical difference was observed regarding the effect of oral health education methods on the oral health of school children with visual impairment. It seems that the ATP technique alone can be an effective method to provide oral health education and improve the oral health status of visually impaired children. The results of this study can guide policymakers to design suitable school-based programs to improve the oral health of visually impaired children.

## Data Availability

The datasets used and analyzed during the current study are available from the corresponding author on reasonable request.

## Abbreviations

ATP: Audio Tactile Performance
OHI-S: Oral Hygiene Index-Simplified
BOP: Bleeding On Probing
SPSS: Statistical Software for Social Sciences
SAS: Statistical Analysis Software
CONSORT: Consolidated Standards of Reporting Trials
ANOVA: Analysis Of Variance
P: P-value
ICC: Intra-class Correlation Coefficient
DI: Debris Index
CI: Calculus Index
95% CI: 95% Confidence Interval
EST: Estimation
SE: Standard Error
OR: Odds Ratio
SD: Standard deviation
Ref: reference category
